# Unravelling Osteoporosis: Key Genes and Potential Therapies

**DOI:** 10.1111/jcmm.70759

**Published:** 2025-08-05

**Authors:** Huichao Fu, Yunjiao Wu, Hongfei Lv, Li‐an Qiu, Weifeng Hu, Hongyun Wu, Junda Qian, Xiongjie Liang, Xiaoyan Wang, Gongping Xu

**Affiliations:** ^1^ Department of Orthopedic Surgery, Second Affiliated Hospital Harbin Medical University Harbin Heilongjiang China; ^2^ Department of Respiratory Medical Oncology Harbin Medical University Cancer Hospital Harbin Heilongjiang China

**Keywords:** drug target, Mendelian randomisation, osteoporosis, transcriptomics

## Abstract

Osteoporosis is a metabolic bone disease characterised by decreased bone mass and increased fracture risk, especially in aging women. Current treatments have limitations and side effects, prompting the need for novel therapeutic targets. Using Mendelian randomisation (MR) on the basis of GWAS data from the FinnGen consortium, we identified druggable genes associated with osteoporosis. Transcriptomic profiling, single‐cell RNA sequencing (scRNA‐seq) and immune infiltration analysis were employed to explore gene expression patterns and immune relevance. Gene set enrichment analysis and gene set variation analysis were used to investigate related signalling pathways. Three genes—FMO4, PSMA4 and VEGFA—were significantly associated with osteoporosis risk. FMO4 showed a protective association and was enriched in vascular and immune cells, suggesting roles in oxidative stress and microenvironment regulation. PSMA4, involved in proteasome activity, was upregulated in macrophages and T cells, potentially influencing bone remodelling through immune‐related protein degradation. VEGFA expression correlated positively with osteoporosis risk, possibly via ER‐β–mediated signalling that promotes osteoblast apoptosis. All three genes were involved in key pathways, including calcium signalling, Wnt/β‐catenin, PI3K/Akt and Hedgehog signalling. Immune analysis revealed strong associations with dendritic cells and macrophages. This study identifies FMO4, PSMA4 and VEGFA as key genes associated with osteoporosis, analyses their molecular mechanisms and regulatory networks and elucidates their relationship with the disease. Furthermore, it suggests 52 candidate compounds potentially interacting with VEGFA and 8 with PSMA4, offering a basis for further investigation into their therapeutic potential.

## 1 Introduction

Because of demographic shifts such as global population aging and increased life expectancy, osteoporosis has become a major public health concern worldwide. The incidence is significantly higher in women than in men, and the number of affected individuals is projected to double between 2010 and 2040 [1]. Osteoporosis is a metabolic bone disease characterised by reduced bone strength and increased fracture risk [2], often leading to a significant decline in quality of life. It is estimated that approximately 20% of individuals aged 50 and older who sustain a hip fracture will die within 1 year, and nearly 50% will experience long‐term disability [3]. Therefore, improving early diagnosis and developing more effective therapeutic strategies are critical.

The pathogenesis of osteoporosis is multifactorial, involving age, sex, genetic susceptibility, medication use and comorbid conditions such as rheumatoid arthritis [4]. Although current treatments–such as bisphosphonates and monoclonal antibodies–are clinically effective, they are associated with limitations and adverse effects, including osteonecrosis of the jaw and atypical femoral fractures [5]. Thus, identifying novel molecular contributors and therapeutic targets is essential to advancing treatment options and minimising complications.

Mendelian randomisation (MR) is a genetic epidemiological approach that uses genetic variants as instrumental variables to assess potential causal relationships between exposures and disease outcomes [6]. Drug target MR, an extension of this method, evaluates variants in or near druggable genes, thereby mimicking the biological effects of pharmacological interventions [7]. This approach has been applied to assess the efficacy and safety of medications such as angiotensin‐converting enzyme inhibitors [8, 9, 10], and is increasingly used in drug discovery and repurposing efforts [9, 11].

Despite its value, conventional MR analyses are largely limited to gene–phenotype associations and often fail to provide mechanistic insight into downstream transcriptional regulation or cell‐type specificity. Furthermore, integrative frameworks combining MR with transcriptomic and single‐cell analyses remain rare. To address these gaps, we developed a comprehensive ‘MR–Transcriptome–Single Cell’ approach that (1) identifies candidate drug targets using FinnGen and EBI GWAS data, (2) maps biological pathways—such as bile acid signalling—regulated by FMO4 through gene set enrichment analysis (GSEA) and (3) locates FMO4 expression specifically in osteoblast precursor cells using single‐cell RNA sequencing data. This integrative strategy aims to enhance the identification of osteoporosis‐related targets and support future therapeutic research.

## 2 Results

### 2.1 Mendelian Randomisation Analysis of the Training Set

**FIGURE 1:**
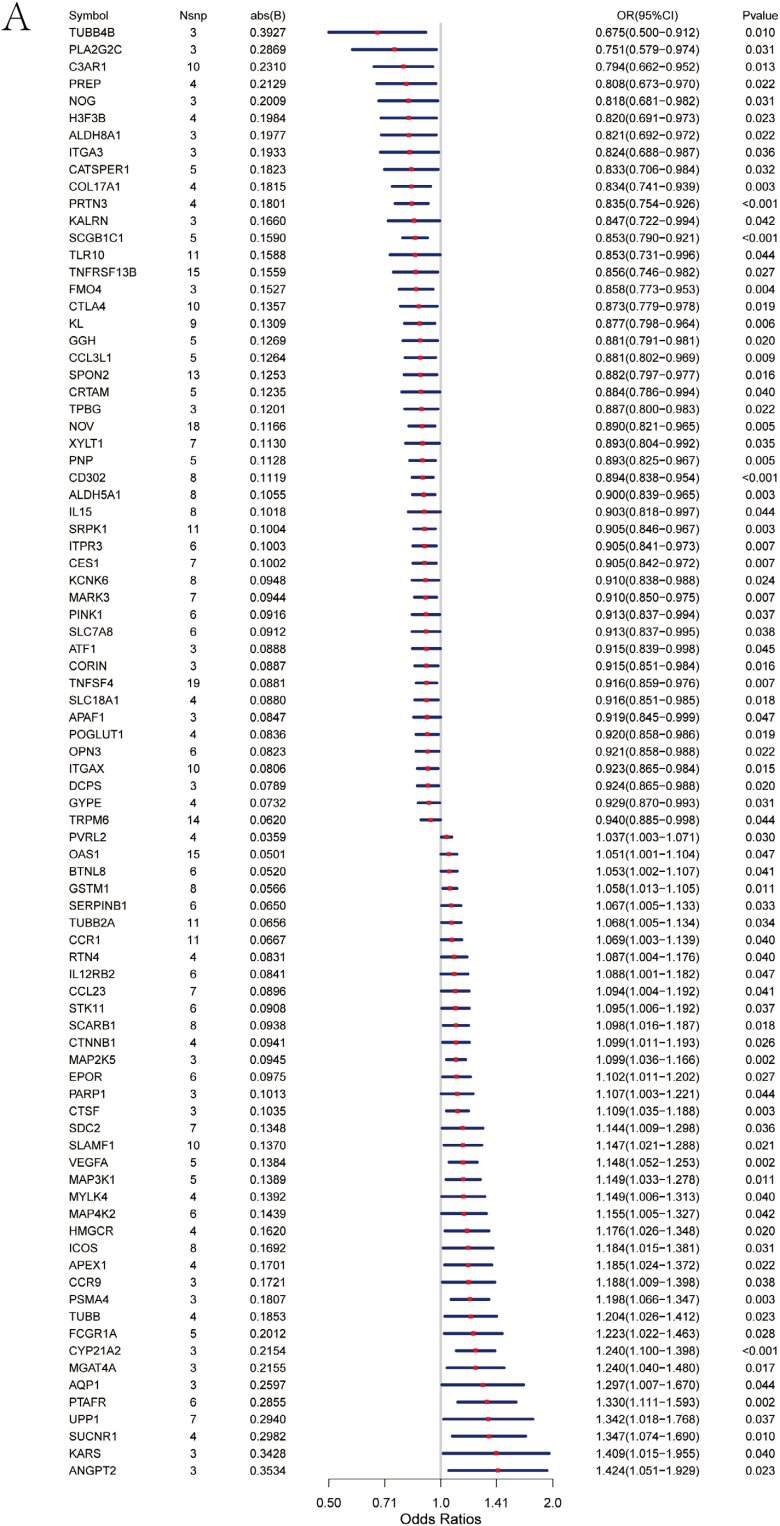
Forest plot of druggable gene–osteoporosis associations on the basis of Mendelian randomisation analysis. Each point represents the odds ratio (OR) and 95% confidence interval (CI) for the association between the genetically predicted expression of a druggable gene and the risk of osteoporosis. Genes are ranked by the absolute value of their effect size abs (B). Horizontal lines represent 95% CIs, and red points indicate the point estimates of ORs. SNP count (Nsnps), effect size and *p* values are shown in accompanying columns. Associations with *p* < 0.05 were considered statistically significant.

We obtained druggable genes from the literature [12]. We obtained osteoporosis‐related GWAS data from the FinnGen biobank to pinpoint key druggable genes affecting osteoporosis. The summary statistics for 399,054 samples (Controls: 391,037; Cases: 8017) were used, with the outcome ID for osteoporosis being finngen_R10_M13_OSTEOPOROSIS. Data for exposure and outcome were extracted using the extract_instruments and extract_outcome_data functions. MR analysis revealed 85 genes linked to eQTL‐positive outcomes (Figure 1, IVW *p* < 0.05). The genes TUBB4B, PLA2G2C, C3AR1, PREP, NOG, H3F3B, ALDH8A1, ITGA3, CATSPER1, COL17A1, PRTN3, KALRN, SCGB1C1, TLR10, TNFRSF13B, FMO4, CTLA4, KL, GGH, CCL3L1, SPON2, CRTAM, TPBG, NOV, XYLT1, PNP, CD302, ALDH5A1, IL15, SRPK1, ITPR3, CES1, KCNK6, MARK3, PINK1, SLC7A8, ATF1, CORIN, TNFSF4, SLC18A1, APAF1, POGLUT1, OPN3, ITGAX, DCPS, GYPE, and TRPM6 are linked to a reduced risk of osteoporosis, whereas PVRL2, OAS1, BTNL8, GSTM1, SERPINB1, TUBB2A, CCR1, RTN4, IL12RB2, CCL23, STK11, SCARB1, CTNNB1, MAP2K5, EPOR, PARP1, CTSF, SDC2, SLAMF1, VEGFA, MAP3K1, MYLK4, MAP4K2, HMGCR, ICOS, APEX1, CCR9, PSMA4, TUBB, FCGR1A, CYP21A2, MGAT4A, AQP1, PTAFR, UPP1, SUCNR1, KARS and ANGPT2 are associated with an increased risk of osteoporosis. Additional sensitivity analysis of the 85 causal relationships indicated that excluding any single SNP did not notably impact the overall error line. This indicates that the 85 causal relationships selected in this study are robust.

### 2.2 MR Analysis and Colocalisation Analysis of the Validation Set

The validation set osteoporosis‐related GWAS summary data were obtained from 667,227 samples (controls: 648,913; cases: 18,314), with the outcome ID being GCST90018887. The data for exposure and outcome were sequentially extracted using the extract_instruments and extract_outcome_data functions.

**FIGURE 2:**
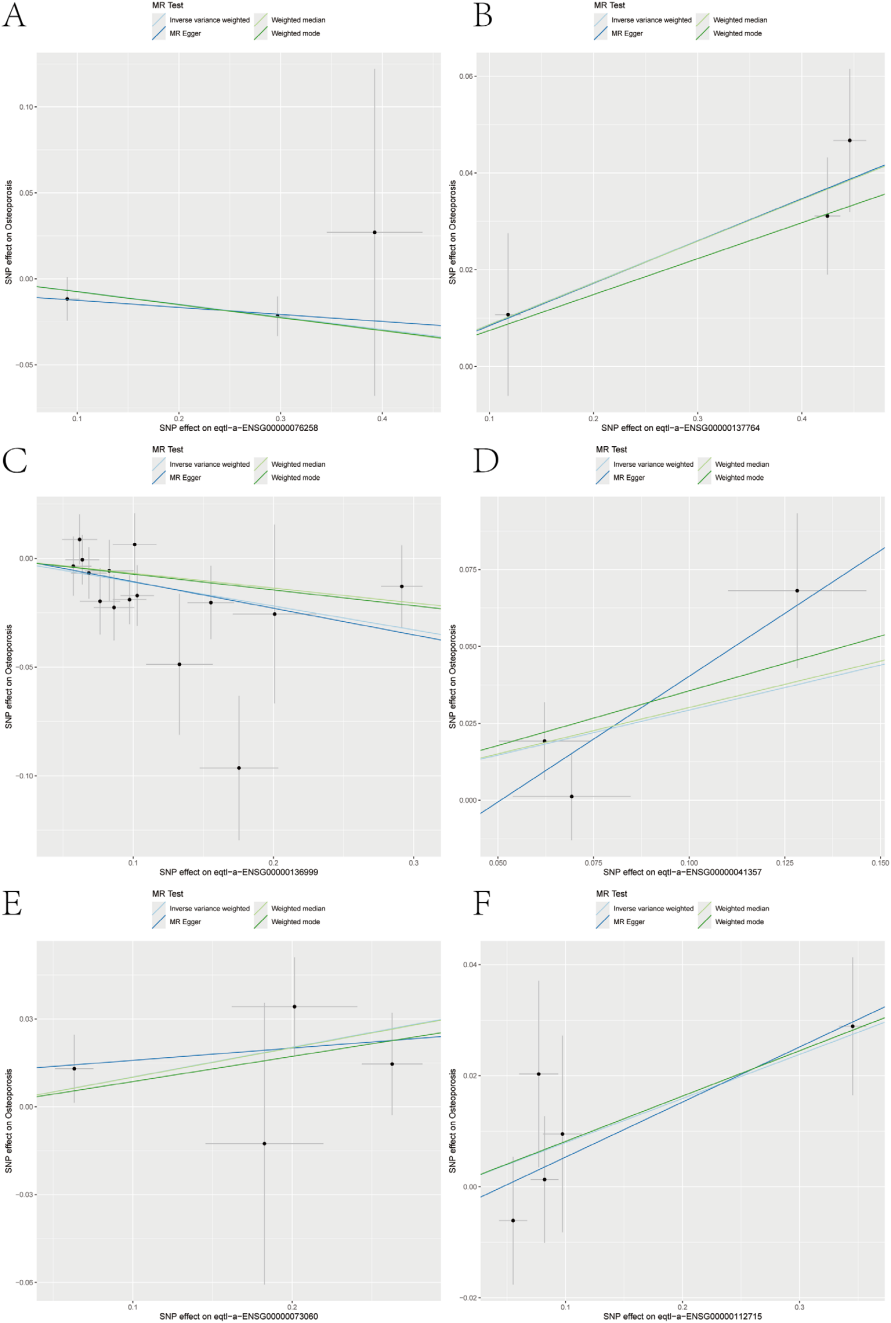
Scatter plots showing MR analysis of selected genes associated with osteoporosis. Scatter plots illustrate the SNP effects on gene expression (x‐axis) and their corresponding effects on osteoporosis (y‐axis). Each plot represents a single gene. Four MR methods are shown: inverse variance weighted (IVW), MR‐Egger, weighted median and weighted mode. Each black dot indicates a single nucleotide polymorphism (SNP) used as an instrumental variable. Regression lines represent causal effect estimates for each MR method. (A–F) Causal effects of genetically predicted expression of six selected genes on osteoporosis risk.

**FIGURE 3:**
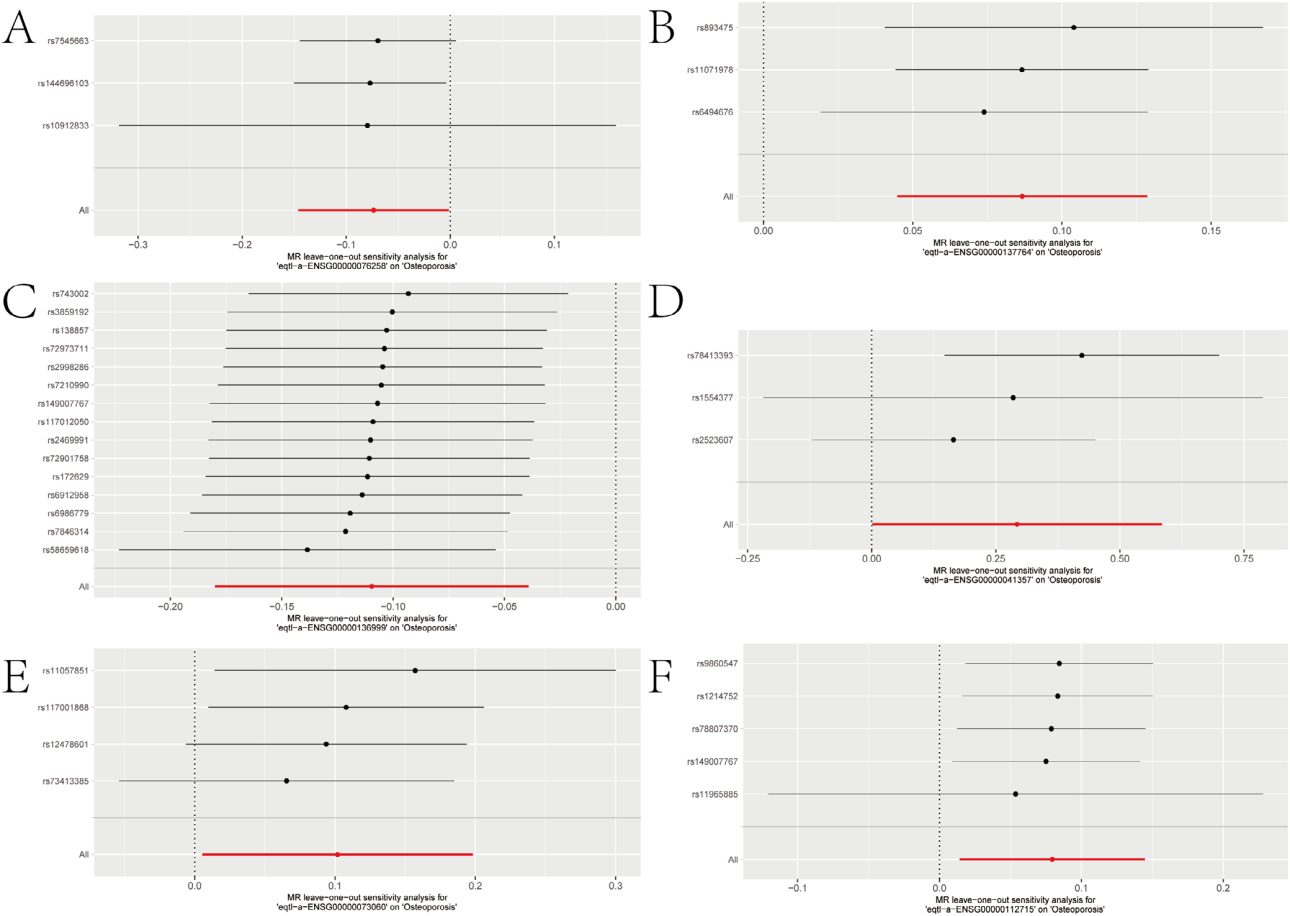
Leave‐one‐out sensitivity analysis for six selected genes in MR. Each plot shows the effect estimate on osteoporosis after sequentially removing each single nucleotide polymorphism (SNP) from the instrument set for a given gene. The black dots represent the estimated causal effect when each corresponding SNP is removed, with horizontal lines indicating 95% confidence intervals. The red dot represents the overall causal estimate using all SNPs. (A–F) Leave‐one‐out plots for six candidate genes, respectively, corresponding to those shown in Figures 2 and 4.

**FIGURE 4:**
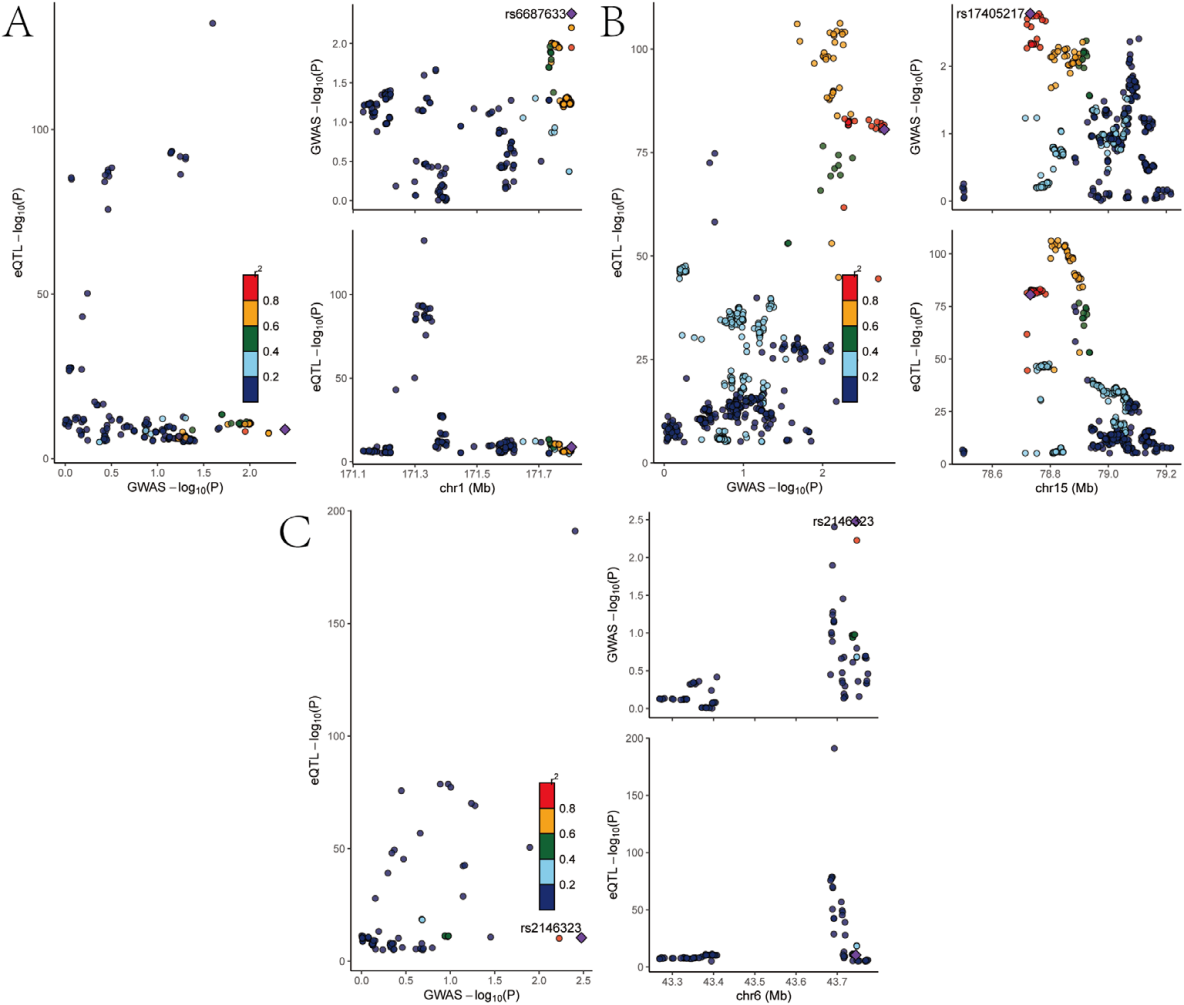
Colocalisation analysis of eQTL and GWAS signals for selected genes. Each panel shows colocalisation plots of eQTL and GWAS signals in the same genomic region. The upper‐left subpanel in each shows a scatter plot of –log_10_ (P) for GWAS versus eQTL associations; colours represent posterior probabilities (PP4) supporting colocalisation. The bottom and right subpanels display the genomic position (in Mb) on the x‐axis and corresponding –log_10_ *p* values for GWAS and eQTL on the y‐axis. The top candidate SNP in each region is highlighted. (A–C) Colocalisation plots for three genes with strong evidence of shared causal variants between eQTL and osteoporosis GWAS signals.

MR analysis revealed six genes linked to eQTL‐positive outcomes (Figure 2A–F, IVW *p*‐value < 0.05). FMO4 and NOV were associated with a decreased risk of osteoporosis, whereas MAP2K5, PSMA4, SCARB1 and VEGFA were linked to an increased risk. Additional sensitivity analysis of the six causal relationships demonstrated their robustness, as excluding any individual SNP did not notably alter the overall error line (Figure 3A–F). The six genes were analyzed for eQTL‐GWAS co‐location. FMO4, PSMA4 and VEGFA showed high probabilities (> 0.75; Figure 4A–C) and were selected as key genes for further analysis.

### 2.3 GSEA

**FIGURE 5:**
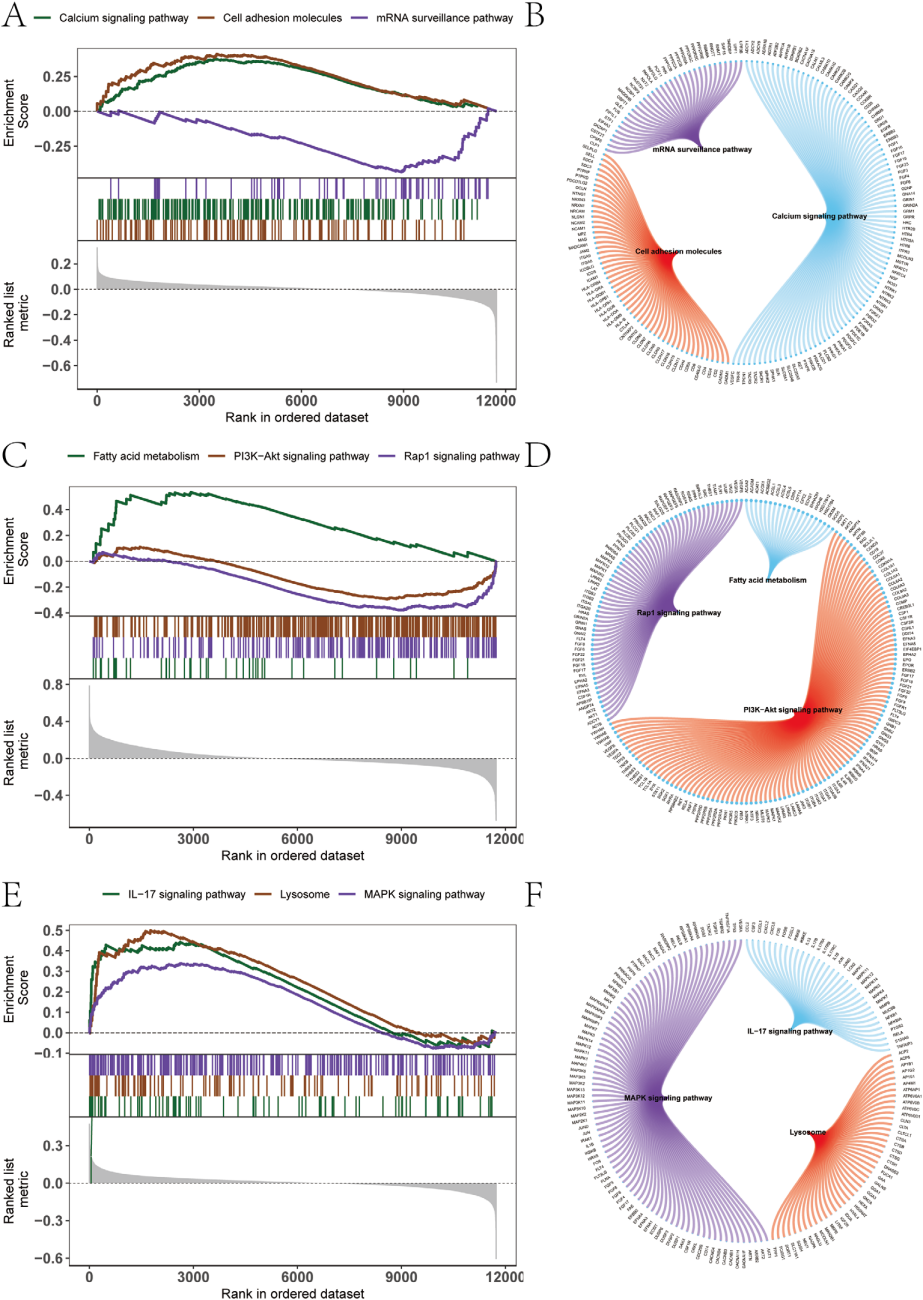
Gene Set Enrichment Analysis (GSEA) and chord diagrams of representative signalling pathways. (A, C, E) GSEA plots showing enrichment scores of hallmark pathways in relation to the ranked gene expression dataset. Top enriched pathways are labelled by colour, including calcium signalling, PI3K‐Akt signalling and IL‐17 signalling among others. The x‐axis represents the ranked genes, and the y‐axis shows the running enrichment score. (B, D, F) Chord diagrams visualising the relationships between core enriched genes and selected KEGG pathways. Genes shared across multiple pathways are represented by arcs connecting them to each respective pathway segment.

We conducted GSEA to investigate the signalling pathways associated with the three key genes. The results indicated that FMO4 was enriched in the mRNA surveillance, calcium signalling, and cell adhesion molecules pathways (Figure 5A,B). PSMA4 was enriched in fatty acid metabolism, Rap1 signalling pathway and PI3K‐Akt signalling pathway (Figure 5C,D). VEGFA was enriched in the IL‐17 signalling pathway, MAPK signalling pathway and lysosome pathway (Figure 5E,F).

### 2.4 Gene Set Variation Analysis (GSVA)

**FIGURE 6:**
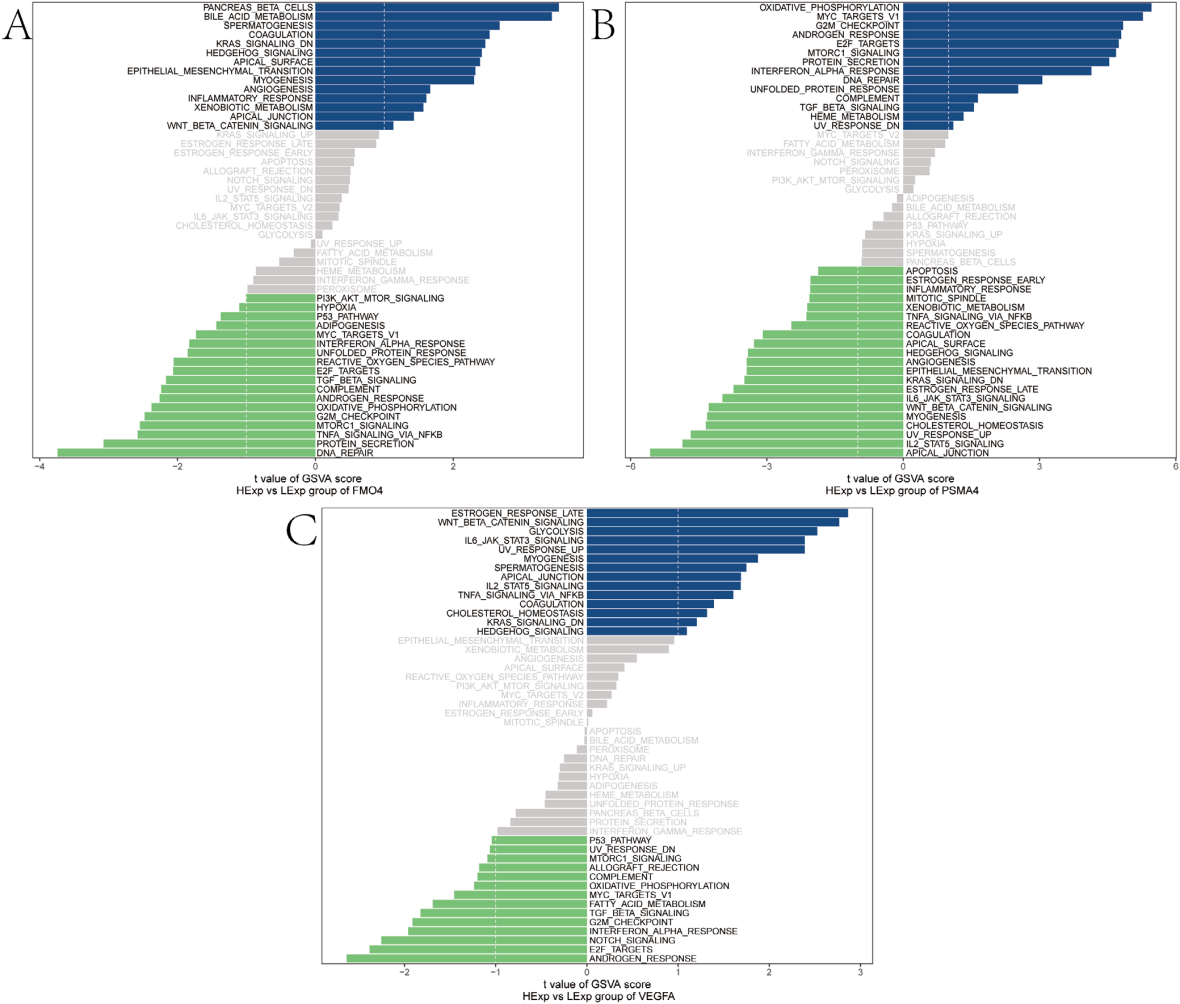
Pathway enrichment analysis on the basis of Gene Set Variation Analysis (GSVA) for high (HE) vs. low expression (LE) groups of key genes. Bar plots display the differential enrichment of hallmark pathways between (HE) and (LE) groups of selected genes on the basis of GSVA scores. The x‐axis shows the *t* value of GSVA scores (HE vs. LE), and the y‐axis lists the significantly enriched pathways. Blue bars represent upregulated pathways in the low‐expression group, green bars indicate those enriched in the high‐expression group, and grey bars show non‐significant changes. (A–C) GSVA‐based pathway enrichment for FMO4 (A), PSMA4 (B) and VEGFA (C).

GSVA indicated that the FMO4 high‐expression group was enriched in bile acid metabolism, hedgehog signalling and Wnt‐beta‐catenin signalling pathways (Figure 6A). PSMA4 was enriched in DNA repair, TGF‐beta signalling and heme metabolism pathways (Figure 6B). VEGFA was involved in the signalling pathways of Wnt‐beta‐catenin, IL‐6/JAK/STAT3 and IL‐2/STAT5 (Figure 6C). The results indicate that pivotal genes might affect disease progression via these signalling pathways. Notably, the observed enrichment of the ‘bile acid metabolism’ pathway may reflect broader systemic metabolic effects rather than bone‐specific alterations. This caveat should be considered when interpreting its relevance to osteoporosis pathophysiology.

### 2.5 Immune Infiltration Analysis

**FIGURE 7:**
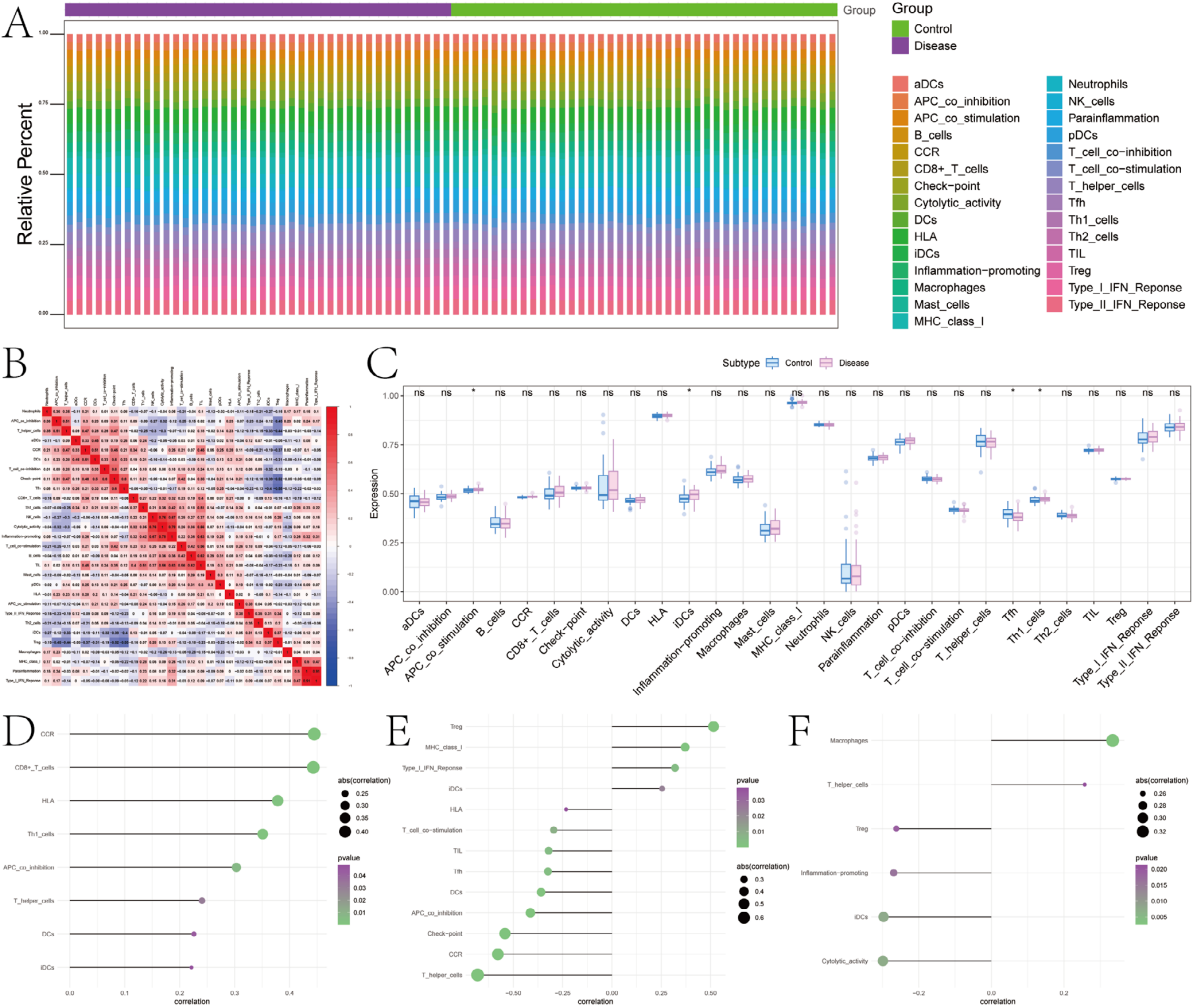
Immune infiltration landscape and correlation analysis between immune cell subsets and disease status. (A) Stacked bar plot showing the relative proportion of immune cell types across control and disease groups. (B) Heatmap of pairwise correlations among immune cell types across all samples. The values in each cell represent Spearman correlation coefficients. (C) Boxplot comparing the expression levels of immune cell signatures between control (blue) and disease (pink) groups. (D–F) Bubble plots showing significant correlations between key immune cell subsets and gene expression (or other phenotype features). The size of each dot indicates the absolute correlation coefficient, and the colour gradient indicates statistical significance (*p*‐value).

The local microenvironment, consisting of immune cells, extracellular matrix components, diverse growth factors, inflammatory mediators and specific physicochemical characteristics, exerts a profound influence on disease diagnosis and sensitivity to clinical treatment. In this study, we systematically analysed the relationship between key genes and immune cell infiltration within an osteoporosis dataset, aiming to further elucidate the potential molecular mechanisms through which these genes contribute to the progression of osteoporosis. Additionally, we characterised the proportional distribution of immune cell populations in each patient and investigated the correlations among different immune cell types (Figure 7A,B). Moreover, statistically significant differences were identified between the two groups in terms of APC co‐stimulation, immature dendritic cells (iDCs), T follicular helper (Tfh) cells and T helper 1 (Th1) cells (Figure 7C).

### 2.6 The Association Between Essential Genes and Immune Cells

We additionally investigated the association between essential genes and immune cells. FMO4 was found to be significantly positively correlated with CCR, CD8^+^ T cells, APC_co_inhibition and T helper cells (Figure 7D). PSMA4 exhibited a notable positive correlation with Treg, MHC class I, and Type I IFN response, while demonstrating a significant negative correlation with T helper cells, CCR and APC_co_inhibition (Figure 7E). VEGFA showed a significant positive correlation with macrophages and T helper cells, while exhibiting a negative correlation with cytolytic activity, DCs and inflammation‐promoting pathways (Figure 7F).

### 2.7 Association Between Essential Genes and Immune Modulators

**FIGURE 8:**
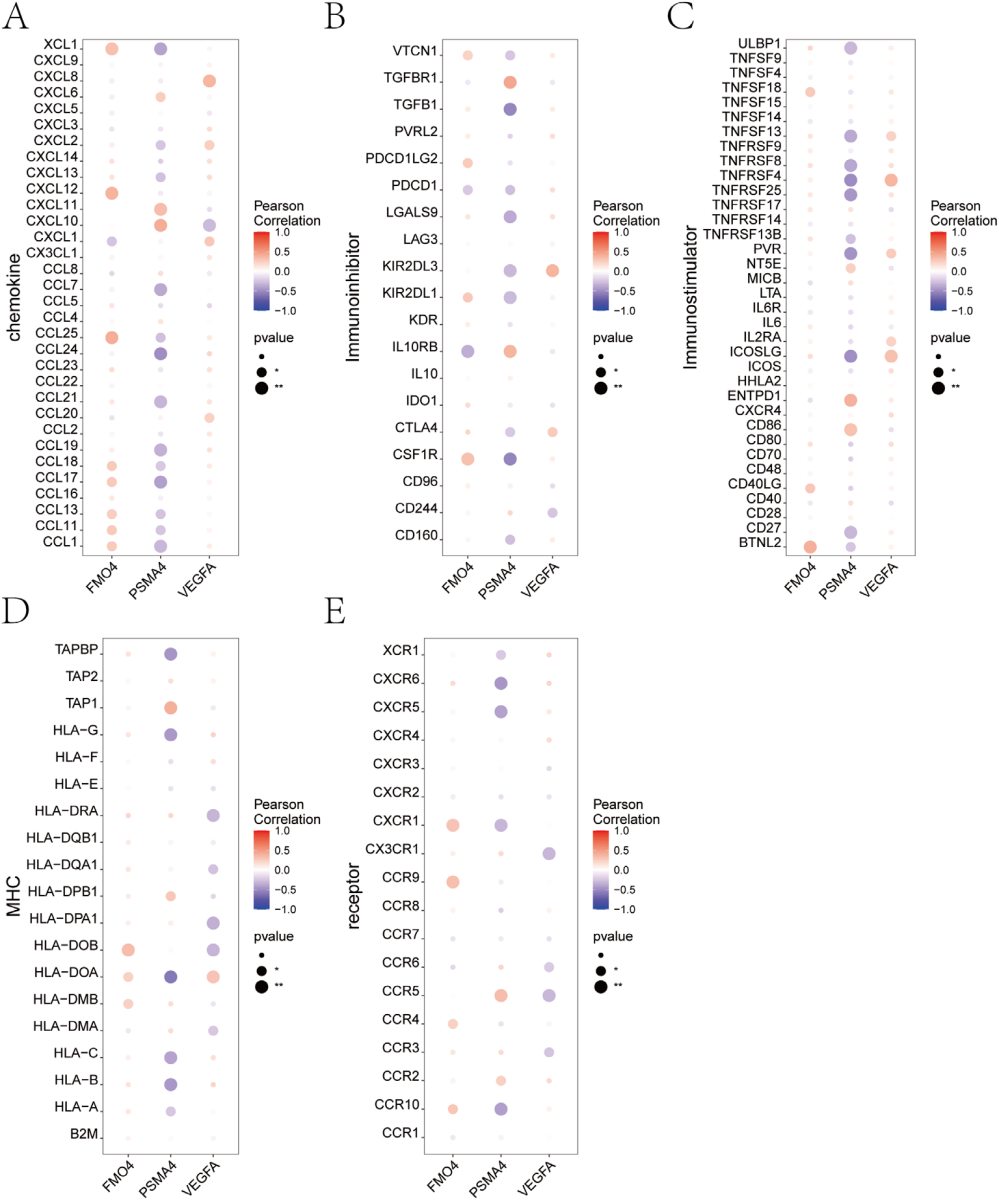
Correlation between key genes and immune‐related gene signatures. Pearson correlation analysis was conducted to explore associations between three key genes (FMO4, PSMA4, VEGFA) and various immune‐related gene sets. The bubble colour indicates the correlation coefficient (from blue = negative to red = positive), and the bubble size reflects the statistical significance (*p*‐value). (A) Correlations with chemokines. (B) Correlations with immune inhibitors. (C) Correlations with immune stimulators. (D) Correlations with MHC‐related genes. (E) Correlations with immune receptors.

We examined the correlation between key genes and diverse immune factors, such as immunosuppressive and immunostimulatory factors, major histocompatibility complex (MHC), chemokines and receptors. These analyses indicate that key genes are significantly linked to immune cell infiltration levels and are essential in the immune microenvironment (Figure 8A–E).

### 2.8 Transcriptional Regulation and miRNA Network Related to Key Genes

**FIGURE 9:**
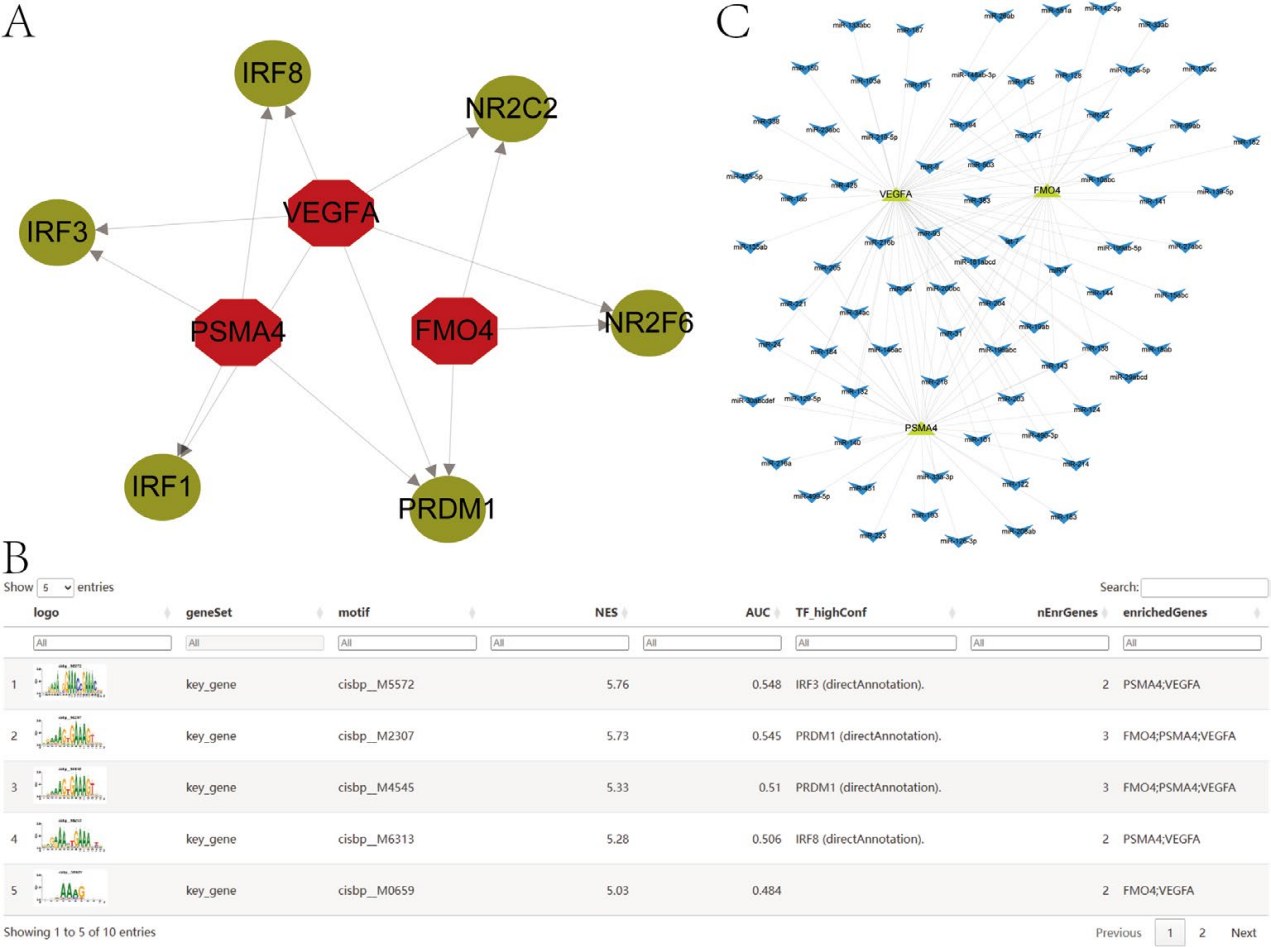
Transcription factor regulatory network analysis of key genes. (A) Predicted upstream transcription factors (TFs) regulating FMO4, PSMA4 and VEGFA, visualised as a directed network. Red nodes represent key genes, and green nodes indicate transcription factors. Arrows denote regulatory direction. (B) Summary of top enriched TF‐binding motifs from motif enrichment analysis. NES = normalised enrichment score; AUC = area under the curve for TF prediction confidence; enrichedGenes = key genes associated with each motif. (C) Global TF–target gene interaction network of FMO4, PSMA4 and VEGFA, showing shared and unique transcriptional regulators.

This study identified that the three key genes are regulated by multiple transcription factors and common mechanisms. Enrichment analysis was performed on these transcription factors using the cumulative recovery curve. Analysis of key genes for motif‐TF annotation and selection identified motif cisbp__M5572 as having the highest normalised enrichment score (NES = 5.76). Figure 9A,B illustrates the enriched motifs and associated transcription factors for the key genes identified in this study. Using the miRcode database, we predicted potential miRNAs for the three key genes, identifying 78 miRNAs and 123 mRNA‐miRNA pairs, which were visualised in Cytoscape (Figure 9C).

### 2.9 Correlation Between Key Genes and Metabolic Pathways and Disease Progression Genes

**FIGURE 10:**
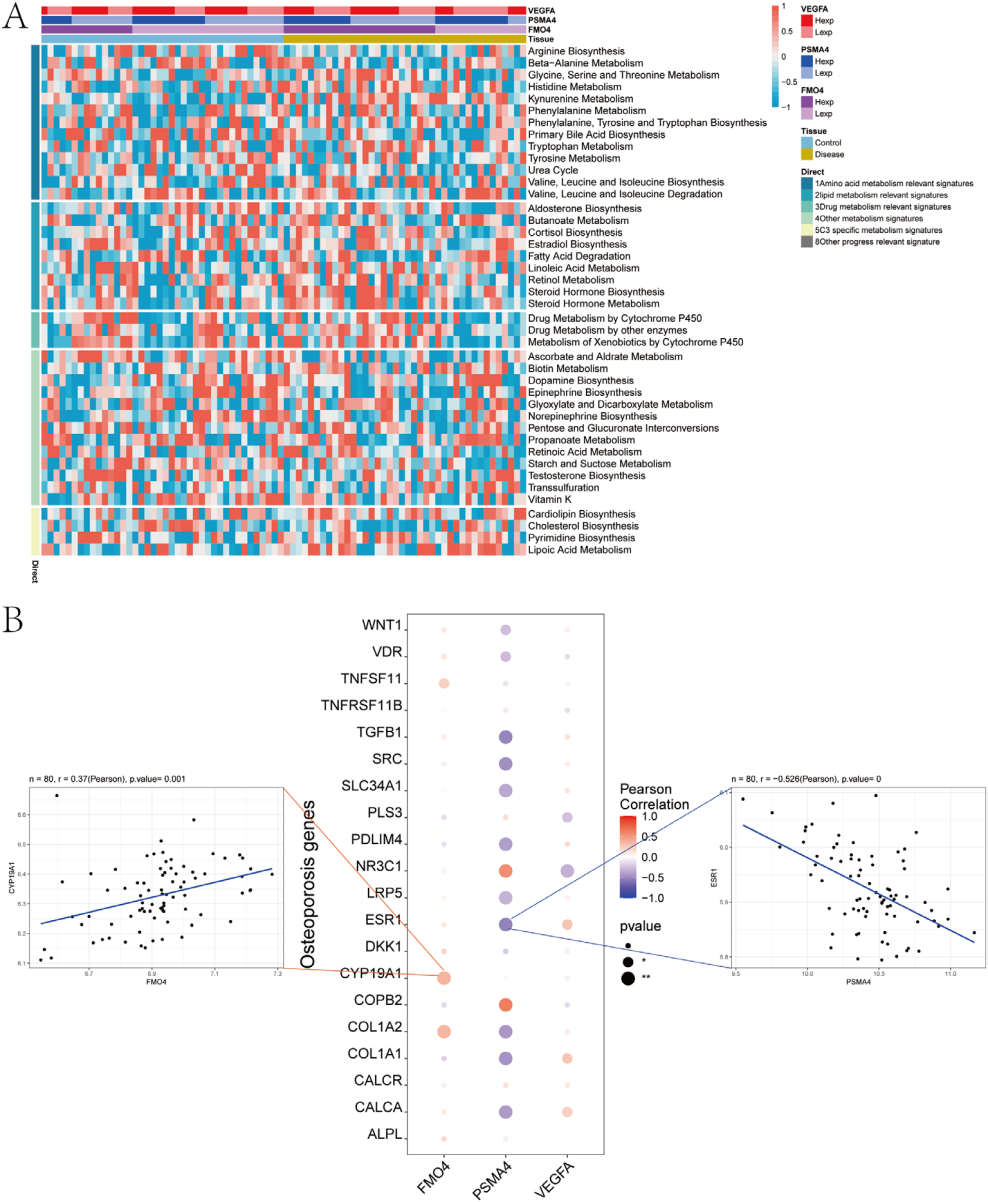
Correlation analysis of key genes with metabolic signatures and osteoporosis‐related genes. (A) Heatmap showing the correlation between the expression of FMO4, PSMA4, and VEGFA and various metabolic pathway gene signatures. Rows represent samples, and columns represent metabolic pathways grouped by biological category. Expression levels of key genes are shown in the top annotation. (B) Bubble plot showing Pearson correlations between key genes and known osteoporosis‐associated genes. The colour represents the direction and strength of correlation, and bubble size indicates statistical significance. Representative correlation plots between FMO4 and CYP19A1, and between PSMA4 and ESR1, are shown as scatter plots with regression lines.

Next, we analysed the correlation between the key genes and metabolic pathways. In the metabolic pathway heatmap, it was observed that the high‐expression group of FMO4 had a significantly higher score in the Drug metabolism relevant signatures category compared to the low‐expression group (Figure 10A). We obtained osteoporosis‐related genes from the GeneCards database (https://www.genecards.org/) and extracted the top 20 genes. A correlation analysis between the expression levels of key genes and disease‐related genes revealed significant associations. FMO4 showed a significant positive correlation with CYP19A1 (cor =0.37), whereas PSMA4 exhibited a significant negative correlation with ESR1 (cor = −0.526) (Figure 10B).

### 2.10 Quality Control and Clustering of Single‐Cell Data

**FIGURE 11:**
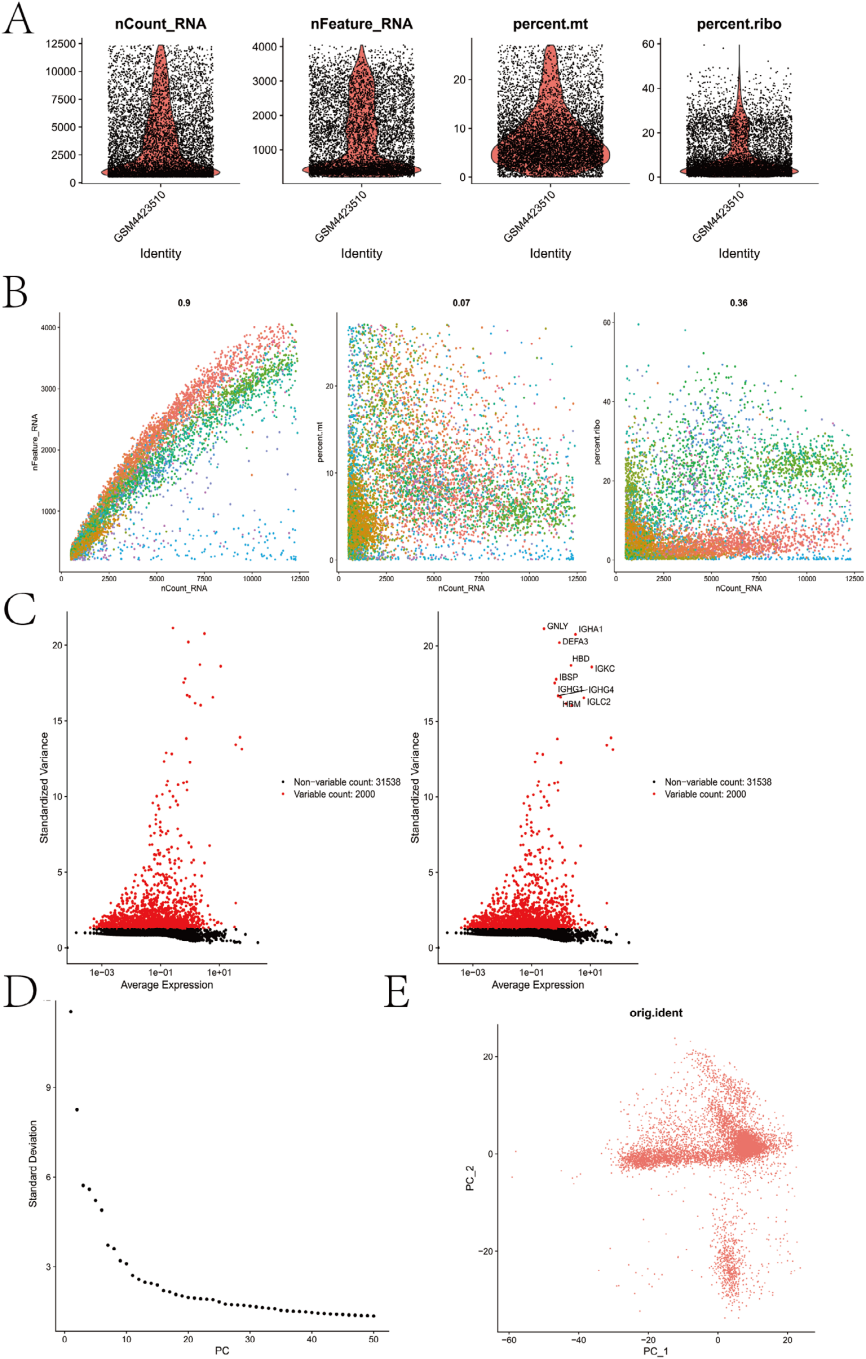
Quality control and preprocessing of single‐cell RNA sequencing data. (A) Violin plots showing distributions of nCount_RNA, nFeature_RNA, percent.mt (mitochondrial gene content) and percent.ribo (ribosomal gene content) in the sample GSM4423510. (B) Correlation plots between nCount_RNA and nFeature_RNA (left), percent.mt (middle) and percent.ribo (right), indicating sample quality characteristics and relationships among key metrics. (C) Selection of highly variable genes on the basis of standardised variance and average expression; 2000 genes were identified as variable for downstream analysis. (D) Elbow plot showing the standard deviation of each principal component, guiding selection of the top PCs for dimensionality reduction. (E) Principal component analysis (PCA) visualisation showing cell distribution across the top two PCs.

Following quality control, 7471 high‐quality cells were retained (Figure 11A,B). These cells exhibited a median gene count > 200 and mitochondrial gene content within physiological ranges. DoubletFinder effectively removed putative doublets, ensuring dataset integrity. HVG analysis identified 2000 genes demonstrating significant expression variability across the cellular landscape (Figure 11C). Dimensionality reduction through PCA (Figure 11D) and batch effect correction using Harmony (Figure 11E) facilitated downstream clustering.

### 2.11 Cell Type Annotation and Key Gene Expression Patterns

**FIGURE 12:**
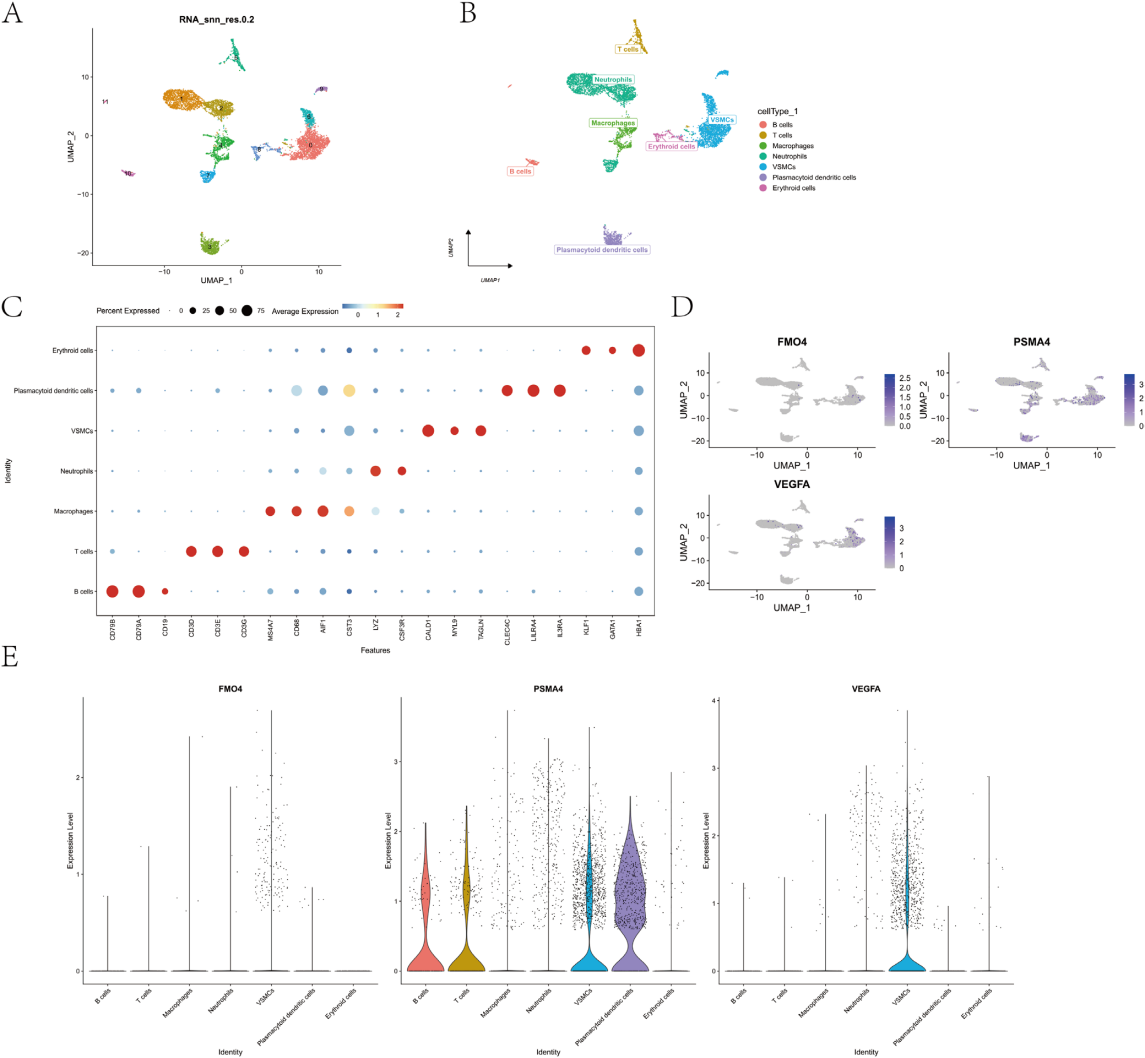
Cell clustering and gene expression profiling on the basis of single‐cell RNA‐seq data. (A) UMAP visualisation of cell clusters on the basis of single‐cell RNA sequencing, coloured by unsupervised clustering (resolution = 0.2). (B) UMAP plot showing annotation of cell types, including T cells, B cells, macrophages, neutrophils, VSMCs, plasmacytoid DCs and epithelial cells. (C) Dot plot showing expression levels and percentage of expressing cells for canonical marker genes across identified cell types. (D) UMAP feature plots displaying spatial expression of key genes (FMO4, PSMA4 and VEGFA) across the entire cell population. (E) Violin plots showing expression distribution of FMO4, PSMA4 and VEGFA in different cell types, highlighting distinct expression patterns.

UMAP projection resolved 12 distinct clusters, which were annotated into seven major cell types: B cells, T cells, macrophages, neutrophils, vascular smooth muscle cells (VSMCs), plasmacytoid DCs and erythroid cells (Figure 12A,B). Marker gene expression validated annotations, with canonical markers (e.g., CD79A for B cells, CD3D for T cells) displaying cell‐type‐specific enrichment (Figure 12C). Key genes (FMO4, PSMA4 and VEGFA) exhibited differential expression patterns across cell types (Figures 12D,E), reflecting lineage‐specific functional roles.

### 2.12 Drug Prediction Using the DGIdb Database

**FIGURE 13:**
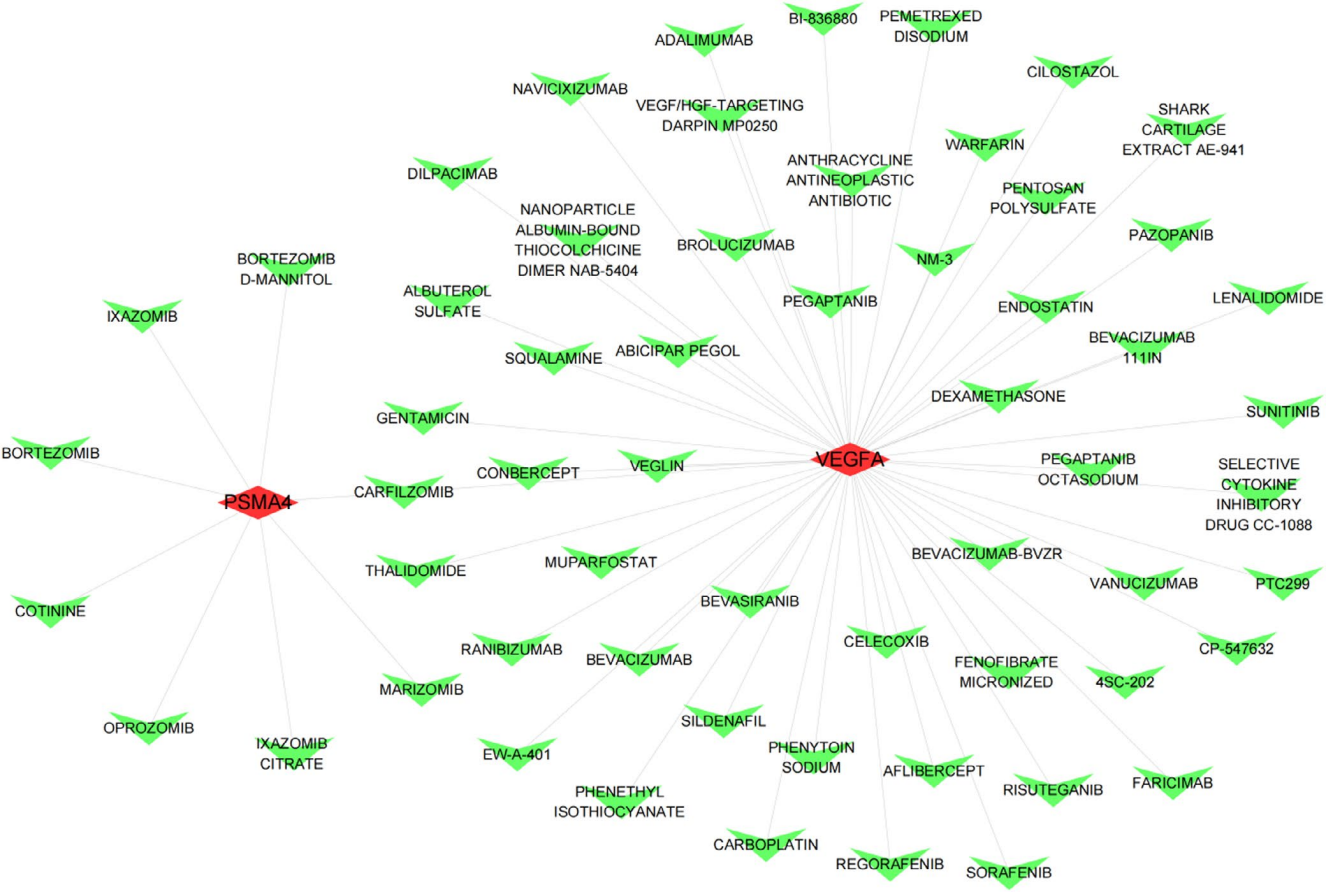
Drug–gene interaction network for PSMA4 and VEGFA. Network visualisation showing drug interactions with two key genes, PSMA4 and VEGFA, identified through database mining. Red nodes represent the target genes, and green nodes represent drugs or compounds. Edges denote known or predicted drug–gene interactions, suggesting potential candidates for further investigation in osteoporosis‐related therapeutic strategies.

This study utilised the DGIdb database to examine potential drug interactions with key genes. Through DGIdb, 52 drugs were identified to interact with VEGFA, and 8 drugs were found to interact with PSMA4. These interactions may provide potential new targets for therapeutic development. Cytoscape was utilised to visualise the drug–gene interactions (Figure 13).

### 2.13 External Validation of the Identified Key Genes

**FIGURE 14:**
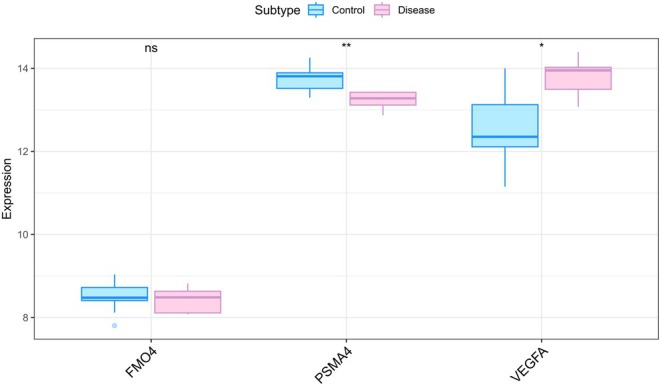
Expression analysis of FMO4, PSMA4 and VEGFA in osteoporosis and control samples from the GSE35959 dataset. The expression levels of three hub genes (FMO4, PSMA4 and VEGFA) were compared between osteoporosis patients (Disease) and healthy controls (Control) using the GSE35959 microarray dataset. FMO4 and PSMA4 were downregulated in the disease group, whereas VEGFA showed upregulated expression, consistent with trends observed in our primary analysis. Statistical significance was assessed using t‐tests: *p < 0.05, **p < 0.01, ***p < 0.001.

To further validate the expression trends of the identified hub genes, we conducted cross‐dataset verification using the GSE35959 transcriptomic dataset, which comprises peripheral blood samples from osteoporosis patients and healthy controls. The analysis revealed that FMO4 and PSMA4 were consistently downregulated in osteoporosis patients, whereas VEGFA exhibited significantly increased expression levels (Figure 14). These findings align with the results from our primary analysis and provide additional support for the potential roles of these genes in the development and progression of osteoporosis.

## 3 Discussion

Osteoporosis is a systemic metabolic bone disease characterised by increased bone fragility and a heightened risk of fractures. It predominantly affects older women, with the prevalence increasing annually [13]. Osteoporosis arises because of an imbalance between bone resorption and formation. Current treatment options include anti‐resorptive and anabolic drugs; however, many of these medications have side effects and require long‐term use, highlighting the urgent need for novel therapeutic approaches to combat osteoporosis.

Large‐scale randomised clinical trials (RCTs) are a key method for evaluating the efficacy of drug treatments and have increasingly become a valuable tool for integrating human genetics with drug development. RCTs have effectively evaluated the impact of HMGCR and PCSK9 inhibitors on coronary artery disease risk [14, 15]. However, RCTs are resource‐intensive and time‐consuming. MR analyses use genetic variants as instrumental variables to determine causal links between exposures and outcomes. Cis‐expression quantitative trait loci (cis‐eQTLs) have been employed to explore the influence of gene expression regulators in diseases such as COVID‐19 and Parkinson's disease [16, 17]. Studies have shown that targeting LDL cholesterol may help prevent and manage abdominal aortic aneurysms, benefiting patients [18].

In this study, we systematically identified VEGFA, PSMA4 and FMO4 as genes significantly associated with osteoporosis risk using MR, and explored their expression patterns using single‐cell RNA sequencing (scRNA‐seq) data from osteoporosis patients. We further investigated their immunological relevance through immune cell infiltration analysis. The integration of genetic, transcriptomic and immunological data provides comprehensive insights into the potential roles of these genes in the pathophysiology of osteoporosis.

FMO4, a member of the flavin‐containing monooxygenase (FMO) family, has not been widely studied in osteoporosis. However, other family members such as FMO3 and FMO5 have been implicated in metabolic and inflammatory regulation—both of which are relevant to bone homeostasis [19]. Although direct evidence linking FMO4 to bone metabolism is currently limited, previous studies have shown that other FMO family members, such as FMO3 and FMO5, play critical roles in the regulation of metabolism and inflammation—processes that may indirectly influence bone homeostasis. Scott et al. [20] have suggested that FMO enzymes may influence oxidative stress and immune signalling, affecting osteoblast and osteoclast activity. Our MR analysis identified a significant inverse association between FMO4 expression and osteoporosis risk, suggesting a protective role. Single‐cell analysis revealed enriched expression of FMO4 in vascular smooth muscle cells (VSMCs) and immune cells, indicating a potential role in regulating redox balance and maintaining bone marrow microenvironment stability, thereby influencing bone remodelling indirectly.

PSMA4, which encodes the α4 subunit of the 20S proteasome core, is a key component of the ubiquitin–proteasome system (UPS). This system is involved in protein degradation, immune regulation, and stress responses. Dysregulated proteasome activity has been linked to abnormalities in osteogenesis and bone metabolism [21]. Clinically, the proteasome inhibitor bortezomib, widely used in multiple myeloma, not only promotes tumour cell apoptosis and suppresses angiogenesis‐related factors but also reduces the invasive effects of myeloma cells on the bone matrix by reversing the hyperactive osteoclast environment, exerting both anti‐myeloma and bone‐protective effects [22]. Given that proteasomal function influences both osteoclast and osteoblast activity, PSMA4 may serve as a regulator of the osteoclastic/osteogenic balance. In support of this hypothesis, our MR analysis revealed a significant association between increased PSMA4 expression and a higher risk of osteoporosis. These findings highlight PSMA4 as a potential contributor to osteoporosis pathogenesis and a candidate for further mechanistic and therapeutic investigation.

VEGFA, a well‐established member of the vascular endothelial growth factor (VEGF) family, encodes a 27‐kDa dimeric glycoprotein critical to angiogenesis and bone remodelling. In addition to promoting neovascularisation, VEGFA acts as a bone anabolic factor, regulating bone formation and resorption to maintain skeletal homeostasis [23, 24, 25]. In our study, elevated VEGFA expression was significantly associated with an increased risk of osteoporosis, suggesting a potential pathogenic role. This finding is consistent with that of Senel et al. [26], who reported significantly lower serum VEGF levels in postmenopausal osteoporosis patients compared to healthy controls, supporting VEGFA's importance in bone metabolism. Moreover, Wu et al. [1] reported that VEGFA expression increases with age. Xu et al. [27] further demonstrated that oestrogen receptor β (ER‐β) upregulates VEGFA, which subsequently activates the downstream c‐JUN/GADD45G signalling axis, leading to osteoblast apoptosis and accelerated bone loss. These observations are in line with our results and collectively suggest that VEGFA plays a critical role in the pathogenesis of osteoporosis. Taken together, these findings indicate that VEGFA may not only serve as a key regulator of bone homeostasis but also represent a promising therapeutic target for future intervention strategies.

Pathway enrichment analyses indicated that FMO4, PSMA4 and VEGFA may influence osteoporosis progression through several critical signalling pathways, including calcium signalling, Wnt/β‐catenin, PI3K/Akt and Hedgehog signalling. Calcium signalling in osteoclasts is essential for regulating differentiation, bone resorption and transcription. Sustained low intracellular calcium levels can activate nuclear factor of activated T‐cells (NFAT), promoting osteoclastogenesis. The Wnt/β‐catenin pathway plays a central role in maintaining bone homeostasis by promoting osteoblast differentiation and preventing osteoclast apoptosis [28, 29]. The PI3K‐Akt‐GSK3β pathway is another important axis in bone disease research. Its activation is closely linked to caspase‐3–mediated apoptosis and can enhance osteoclast activity [30]. The Hedgehog pathway, in cooperation with bone morphogenetic proteins (BMPs), regulates the differentiation of mesenchymal stem cells into osteoblasts and plays a key role in skeletal development and bone maintenance.

Immune infiltration analysis revealed significant correlations between the three genes and critical immune cells, particularly DCs and macrophages. These immune populations are known to modulate inflammation, bone resorption and remodelling, reinforcing the immunomodulatory relevance of FMO4, PSMA4 and VEGFA in osteoporosis pathophysiology.

A notable strength of this study lies in its focus on druggable genes, which enhances the clinical and translational value of the findings. By integrating genetic, transcriptomic and immunologic data, we identified FMO4, PSMA4 and VEGFA as promising therapeutic targets. Additionally, several small‐molecule inhibitors targeting these genes are currently under development, laying the groundwork for future drug discovery and repurposing.

Nevertheless, this study has limitations. First, the genetic effect sizes used in MR are generally modest, and the estimated causal effects reflect lifelong exposure, which may not directly translate to the outcomes of short‐term therapeutic interventions. Second, the GWAS data used in this study predominantly derive from individuals of European descent, which may limit the generalisability of the findings to other ethnic populations. Future studies should aim to replicate these findings in larger and more diverse cohorts and conduct experimental validation to clarify the biological mechanisms involved.

## 4 Conclusions

This study identified key genes linked to osteoporosis, such as FMO4, PSMA4 and VEGFA, and explored their potential molecular mechanisms and regulatory networks. These findings offer valuable insights into the pathological mechanisms underlying osteoporosis and suggest potential candidate genes and pathways for further investigation in drug development and therapeutic research.

## 5 Experimental Section

### 5.1 Data Download

1. Exposure Data: The eQTL data were retrieved from the eQTLGen Consortium database (https://www.eqtlgen.org). The eQTLGen Consortium seeks to clarify the genetic structure influencing blood gene expression and enhance comprehension of the genetic foundations of complex traits. The eQTLGen project, now in its second phase, aims to perform extensive genome‐wide meta‐analyses of blood expression data.
2. Outcome Data: The outcome‐related GWAS data included participants of predominantly European ancestry. The outcome summary data for the training set were obtained from the FinnGen biobank database (finngen_R10_M13_OSTEOPOROSIS). In the FinnGen study, cases were identified using relevant ICD codes. Specifically, the osteoporosis cohort comprised 8017 cases and 391,037 controls. The test set outcome summary data were sourced from the EBI database (GCST90018887). In the EBI study, cases were similarly defined using ICD codes, with the osteoporosis cohort consisting of 18,314 cases and 648,913 controls.
3. The Gene Expression Omnibus (GEO) database, maintained by the National Center for Biotechnology Information (NCBI), is available at https://www.ncbi.nlm.nih.gov/geo/info/datasets.html. The GEO database is a comprehensive resource for gene expression data. Osteoporosis gene expression data were obtained from the GEO database, specifically from dataset GSE56815, annotated with the GPL96 platform. This dataset includes 40 control samples and 40 disease samples and was used to explore the bioinformatic mechanisms of key genes in osteoporosis.
4. The Gene Expression Omnibus (GEO), a public repository maintained by the National Center for Biotechnology Information (NCBI), was utilised to retrieve the single‐cell RNA sequencing (scRNA‐seq) dataset GSE147287, which comprises profiling data from a single osteoporosis patient.

### 5.2 MR Analysis

The outcome‐related GWAS IDs were extracted from the GWAS summary data (https://gwas.mrcieu.ac.uk/) for causal relationships in eQTLs. SNPs with significant associations (*p* < 1e‐5) for each gene across the genome were chosen as potential instrumental variables (IVs). SNPs with R^2^ < 0.001 (clumping window size = 10,000 kb) were considered, and weak instrumental variables were excluded by applying an F‐value >10 filter. Four statistical methods were employed to assess causal relationships: Inverse Variance Weighted (IVW), which combines each SNP's Wald estimate using meta‐analysis; MR‐Egger, relying on the Instrument Strength Independent of Direct Effects (InSIDE) assumption; Weighted Median, permitting accurate causal inference even if up to 50% of instrumental variables are invalid; and Weighted Mode, offering greater power than MR‐Egger with reduced bias and lower type I error rates. The Wald ratio method was applied when a single SNP was implicated in the causal relationship. These methods estimated the impact of cis‐acting and certain trans‐acting gene expressions in whole blood on osteoporosis.

### 5.3 Sensitivity Analysis

A leave‐one‐out sensitivity analysis was conducted using MR to evaluate the impact of individual genetic variants on osteoporosis risk. This method sequentially omits each SNP and recalculates the aggregate effect size of the remaining SNPs to detect and eliminate variants that excessively influence the overall estimate. Removing each SNP provides a new point estimate and its 95% confidence interval, allowing assessment of each SNP's unique contribution and the robustness of the results. The summary included both the overall estimate with all SNPs and the estimates after each SNP was excluded. Comparing these estimates allowed us to assess the robustness of our findings by observing the impact of removing individual SNPs on the overall results.

### 5.4 Colocalisation Analysis

Colocalisation analysis was performed using the coloc method, with eQTL summary data and the GWAS data for osteoporosis. A 100‐kilobase region surrounding each index SNP was used to compute posterior probabilities. In the coloc results, H3 represents the posterior probability that gene expression and osteoporosis are linked but have separate causal variants, whereas H4 denotes the probability that they are associated and share a common causal variant. A coloc threshold of SNP.PP.H4 > 0.75 was used to identify shared causal variants.

### 5.5 GSEA

Patients were categorised into high and low expression groups for GSEA on the basis of gene expression levels. Pathway differences between the two groups were analysed using GSEA. The analysis utilised a background gene set sourced from the MsigDB database, offering annotated gene sets for pathways specific to subtypes. Pathway differential expression between subtypes was examined, ranking the most significantly enriched gene sets (adjusted P‐values < 0.05) by their consistency scores. GSEA is commonly used for investigating the biological significance of disease subtypes and their associated pathways.

### 5.6 GSVA

GSVA is an unsupervised, non‐parametric approach for assessing gene set enrichment in transcriptomic datasets. GSVA transforms gene‐level changes into pathway‐level variations by assigning a comprehensive score to each gene set of interest, thereby assessing the biological functions of samples. Gene sets from the Molecular Signatures Database (MSigDB, version 7.0) were analysed using the GSVA algorithm to calculate enrichment scores, facilitating the evaluation of potential biological function variations across samples.

### 5.7 Immune Cell Infiltration Analysis

The single‐sample Gene Set Enrichment Analysis (ssGSEA) method is widely used to evaluate immune cell types in the microenvironment. This method identifies 29 distinct human immune cell phenotypes, such as T cells, B cells, and NK cells. This study utilised ssGSEA to quantify immune cell infiltration from expression profiles, allowing inference of the relative proportions of 29 immune cell types in the samples.

### 5.8 Transcriptional Regulation Analysis of Important Genes

Transcription factor prediction was conducted using the R package ‘RcisTarget.’ RcisTarget's computations rely on motif analysis. The NES for each motif is influenced by the overall count of motifs in the database. Beyond the annotated motifs, additional annotation files were derived using motif similarity and gene sequence analysis. Estimating motif overexpression in gene sets begins with calculating the area under the curve (AUC) for each motif–motif set pair. This is done by calculating the recovery curve on the basis of the ranking of motifs in the gene set. The NES for each motif is calculated on the basis of the AUC distribution of all motifs within the gene set.

### 5.9 miRNA Network Construction

MicroRNAs (miRNAs) are small non‐coding RNAs that control gene expression by facilitating mRNA degradation or blocking mRNA translation. We investigated miRNAs linked to essential genes to determine their role in regulating gene transcription or degradation. Key gene miRNA data were sourced from the miRcode database, and a miRNA‐gene network was visualised with Cytoscape software.

### 5.10 Prediction and Analysis of Drug Interactions With Key Genes

The Drug‐Gene Interaction Database (DGIdb) serves as an extensive resource detailing established and possible gene‐drug interactions. The dataset includes over 14,000 drug‐gene interactions, involving 2600 genes and 6300 drugs, along with 6700 additional genes. By focusing on key genes, we identified potential therapeutic drugs and visualised the gene‐drug interaction network using Cytoscape software.

### 5.11 Quality Control of Single‐Cell Data

Initial preprocessing was conducted using the Seurat package. Cells were filtered on the basis of criteria including total unique molecular identifier (UMI) counts, number of detected genes per cell, and mitochondrial gene expression proportion (defined as the percentage of total gene expression attributable to mitochondrial genes). Aberrant cells exhibiting mitochondrial gene proportions exceeding three median absolute deviations (MADs) from the median were excluded to remove low‐quality or dying cells. DoubletFinder v2.0.4 was employed to identify and eliminate doublet cells, ensuring dataset purity.

### 5.12 Dimensionality Reduction, Clustering and Cell Annotation

Cells underwent global normalisation using the LogNormalize method, scaling gene expression to a target sum of 10,000 transcripts per cell followed by log transformation. Cell cycle scores were computed using CellCycleScoring and highly variable genes (HVGs) were identified via FindVariableFeatures. Confounding factors (e.g., mitochondrial/ribosomal gene expression, cell cycle phase) were regressed out using ScaleData. Principal component analysis (PCA) was applied for linear dimensionality reduction, with principal components (PCs) selected for subsequent analysis. Batch effects were mitigated using the Harmony algorithm, and non‐linear dimensionality reduction was achieved via Uniform Manifold Approximation and Projection (UMAP). Cell types were annotated by integrating marker genes from CellMarker, PanglaoDB, and published literature, supplemented by automated annotation using the SingleR package.

### 5.13 External Validation of the Identified Key Genes

To validate the expression patterns of the hub genes in an independent cohort, we analysed the publicly available microarray dataset GSE35959 (platform: GPL570), which contains peripheral blood samples from osteoporosis patients (*n* = 5) and healthy controls (*n* = 14). The raw data were retrieved from the Gene Expression Omnibus (GEO) database and preprocessed using R software (v4.3.0). Background correction and quantile normalisation were performed using the limma package. Expression values of FMO4, PSMA4 and VEGFA were extracted for comparison. Group differences were assessed using an unpaired Student's t‐test, and results were visualised as box plots generated with the ggplot2 package. Statistical significance was defined as *p* < 0.05.

### 5.14 Statistical Analysis

To ensure the validity of the MR analysis, we adhered to the three core assumptions: (1) relevance, where instrumental variables (IVs) are strongly associated with the exposure; (2) independence, indicating that IVs are not associated with confounders; and (3) exclusion restriction, meaning that IVs affect the outcome only through the exposure. Violation of the third assumption may indicate the presence of horizontal pleiotropy.

To assess potential horizontal pleiotropy, we performed MR‐Egger regression. A statistically significant non‐zero intercept suggests the presence of directional pleiotropy and potential bias in the causal estimate. In addition, Cochran's Q test was used to evaluate heterogeneity among SNP‐specific causal estimates. A P‐value greater than 0.05 for the Q statistic was considered indicative of no significant heterogeneity.

To further evaluate the robustness of the results and identify any influential single nucleotide polymorphisms (SNPs), a leave‐one‐out sensitivity analysis was conducted. This analysis iteratively removes one SNP at a time to assess its impact on the overall causal estimate.

All statistical analyses were conducted using R software (version 4.3.0). All tests were two‐sided, and a P‐value of less than 0.05 was considered statistically significant.

## Author Contributions

**Huichao Fu:** conceptualization (lead), data curation (supporting), formal analysis (equal), investigation (lead), writing – original draft (lead), writing – review and editing (lead). **Yunjiao Wu:** conceptualization (equal), data curation (equal), formal analysis (equal), investigation (equal), writing – original draft (equal). **Hongfei Lv:** methodology (equal), software (equal). **Li‐an Qiu:** methodology (equal), project administration (equal), resources (equal). **Weifeng Hu:** resources (equal), software (equal). **Hongyun Wu:** methodology (equal), project administration (equal). **Junda Qian:** resources (equal), software (equal). **Xiongjie Liang:** investigation (equal), methodology (equal), project administration (equal). **Xiaoyan Wang:** resources (equal), software (equal). **Gongping Xu:** conceptualization (equal), writing – original draft (supporting), writing – review and editing (supporting).

## Conflicts of Interest

The authors declare no conflicts of interest.

## Data Availability

All data are available in databases. EQTL data were obtained from the eQTLGen consortium database. Osteoporosis GWAS data were obtained from FinnGen biobank and GWAS catalogue database. Osteoporosis microarray data were obtained from GSE56815.
